# Extreme Periodic Fever, Aphthous Stomatitis, Pharyngitis, Adenitis (PFAPA): a discrete group of patients

**DOI:** 10.1186/s12969-023-00880-1

**Published:** 2023-09-01

**Authors:** Mor Broide, Yoel Levinsky, Rotem Tal, Liora Harel, Shoval Shoham, Sabreen Abu Ahmad, Yonatan Butbul Aviel, Gil Amarilyo

**Affiliations:** 1 Department of Pediatrics A, Schneider Children’s Medical Center of Israel, Petach Tikva, Israel; 2 Pediatric Rheumatology Unit, Schneider Children’s Medical Center of Israel, Petach Tikva, Israel; 3 Faculty of Medicine, Tel Aviv University, Tel Aviv, Israel; 4 Schneider Children’s Medical Center of Israel, Petach Tikva, Israel; 5 Department of Pediatrics B, Ruth Rappaport Children’s Hospital, Rambam Health Care Campus, Haifa, Israel; 6 Pediatric Rheumatology Service, Ruth Rappaport Children’s Hospital, Rambam Health Care Campus, Haifa, Israel; 7 The Ruth and Bruce Rappaport Faculty of Medicine, Technion-Israel Institute of Technology, Haifa, Israel

**Keywords:** PFAPA, Corticosteroids, Colchicine

## Abstract

**Objective:**

Periodic fever, aphthous stomatitis, pharyngitis, and cervical adenitis (PFAPA) syndrome is the most common periodic fever syndrome in children; by definition, episodes occur every 2 to 8 weeks. However, in a subset of our patients, we noticed a higher frequency of attacks, of less than 2 weeks, which we refer to as extreme PFAPA (ePFAPA). This group consisted of patients who were extreme upon presentation of PFAPA, and those who became extreme after initiation of abortive corticosteroid treatment. We aimed to characterize demographic and clinical features of ePFAPA, including the two groups, and to compare them to patients with non-extreme PFAPA (nPFAPA).

**Study design:**

: The medical records of 365 patients with PFAPA who attended Schneider Children’s Medical Center of Israel from March 2014 to April 2021 were reviewed. Patients with concomitant familial Mediterranean fever were excluded. Characteristics of the ePFAPA (including subgroups) and nPFAPA groups were compared using Wilcoxon rank sum, Pearson’s chi-squared, and Fisher’s exact tests.

**Results:**

Forty-seven patients (12.9%) were identified as having ePFAPA. Among patients with ePFAPA, compared to patients with nPFAPA, the median (interquartile range) age at disease onset was earlier: 1.5 years (0.7–2.5) vs. 2.5 years (1.5-4.0), P < 0.001; and diagnosis was younger: 2.6 years (2.0-3.6) vs. 4.5 years (3.0-6.2), P < 0.001. A higher proportion of patients with ePFAPA than nPFAPA were treated with colchicine prophylaxis (53% vs. 19%, P < 0.001), but symptoms and signs during flares did not differ significantly between these groups. Demographic and clinical characteristics were similar between patients with ePFAPA from presentation of PFAPA (22, 47% of those with ePFAPA) and ePFAPA from after corticosteroid treatment.

**Conclusion:**

About half the patients categorized with ePFAPA syndrome already had extreme features upon presentation. Patients with ePFAPA compared to nPFAPA presented and were diagnosed at an earlier age.

**Supplementary Information:**

The online version contains supplementary material available at 10.1186/s12969-023-00880-1.

## Introduction

The autoinflammatory syndrome of periodic fever, aphthous stomatitis, pharyngitis, and adenitis (PFAPA) was first reported by Marshall et al. [1]. Symptoms include recurrent episodes of fever together with pharyngitis, tonsillitis, oral aphthae, cervical adenitis, and occasionally, abdominal pain and arthralgia [2–4].

A typical PFAPA flare lasts 3–7 days, and intervals of 2–8 weeks have been reported.Occasionally, flares may occur at predictable intervals [5].

The etiology of the syndrome is unclear, although certain risk factors have been identified, as has a familial tendency to PFAPA [6–8]. Moreover, our group has reported a higher prevalence of PFAPA among children of Mediterranean ancestry, which suggests a genetic predisposition [9], and concomitant presentation with familial Mediterranean fever (FMF) [10]. Indeed, among individuals with PFAPA in Turkey, symptom onset was at a younger age, fever attacks were of shorter duration, and pharyngitis was more frequent, compared to patients from the US [11].

Abortive treatment with glucocorticosteroids quickly resolves the fever [2, 9], although in about half the patients, it also appears to shorten the interval between flares [12].

PFAPA is considered a benign disease. In long-term follow-up, most patients with PFAPA experience spontaneous resolution of symptoms without sequelae [4]. However, the febrile episodes have shown to have a major impact on quality of life of children with PFAPA [13].

According to the literature, the interval between flares of PFAPA is at least 2 weeks [2]. For this study, we defined a subgroup of patients with extreme PFAPA (ePFAPA), as having PFAPA flares with intervals of less than two weeks apart for a minimum of three consecutive months. The aim of this study was to identify and characterize demographic and clinical features of patients with ePFAPA, and to compare them to those of patients with non-extreme PFAPA (nPFAPA).

## Methods

Data were collected retrospectively from the electronic medical charts of all of the children diagnosed with PFAPA, who were followed at the outpatient pediatric rheumatology clinic of Schneider Children’s Medical Center of Israel between March 2014 and April 2021.

The diagnosis of PFAPA was based on previously established clinical criteria [14, 15]. The first of these is periodic fever for at least 6 months with a daily fever of at least 38.5 °C (axillary) for 2 to 7 days. A second criterion is at least five regularly recurring fever episodes with a maximum 2 months interval between them. A third criterion is the presentation of pharyngitis, cervical adenitis, or oral aphthae, at least one in every episode and at least 2 of three in the majority of episodes. A fourth criterion is exclusion of patients with other causes of recurrent fever, infections, immunodeficiency and cyclic neutropenia, according to their medical histories, physical examinations and lab work Additionally, disease onset is before age 6 years, recovery between episodes is complete, and linear growth is normal.

Children with a diagnosis of another periodic fever disease, such as FMF or another rheumatic disease, and children treated with corticosteroids due to other reasons, were excluded. The study was approved by the Research Ethics Board of Rabin Medical Center (approval no. RMC-19-0731).

Routinely during the study period, patients with PFAPA or their parents were requested to report, at each clinical visit, the frequency and characteristics of attacks, and response to therapy, as recorded in a symptom’s diary. PFAPA attacks were identified according to classical symptoms and complete resolution of fever within hours of steroid treatment. Responses to steroids and symptoms differentiated between PFAPA flairs and upper respiratory tract infections. Demographic, genetic, clinical, and treatment data were collected. Patients were classified as having ePFAPA or nPFAPA according to the frequency of attacks. Our definition of ePFAPA included patients with intervals between flares of less than two weeks, for a minimum of three consecutive months, at any time period since disease onset. Hence, the definition of ePFAPA included both patients who started their disease with short intervals (ePFAPA upon presentation) and patients who met the criteria for ePFAPA during the course of their disease. The latter occurred after initiation of a single dose of treatment with glucocorticoids during an attack (erPFAPA, extreme responder PFAPA). In a sub-analysis, we compared characteristics between the three groups: nPFAPA, ePFAPA upon presentation, and erPFAPA. In addition, to verify the status of attacks in the 6 months preceding conduct of the study, parents were contacted via phone calls in April 2021. After granting informed consent, parents were asked to either answer verbally or to complete the study questionnaire (supplementary data) using Research Electronic Data Capture (REDCap) tools hosted at Schneider’s Children Medical Center of Israel [16, 17]. Missing variables were omitted from the final calculations after verifying that omission would not cause clinical or statistical bias.Statistical analysis was performed using IBM-SPSS v 25 for Windows (IBM, Armonk, New York). Descriptive statistics are reported as medians and interquartile ranges (IQRs) or as numbers and percentages. The ePFAPA and nPFAPA groups were compared using Wilcoxon rank sum, Pearson’s chi-squared and Fisher’s exact tests. The significance level was set at 0.05.

## 1. Results

A total of 365 patients with PFAPA were included in the study. Table 1 summarizes the demographic and family history data of the 47 (12.9%) who were classified as having ePFAPA and the 318 patients with nPFAPA. Overall, 204 (56%) were males, 173 in the nPFAPA group (54%) and 31 (66%) in the ePFAPA group (P = 0.14).

**Table 1:** Comparison of demographic data and family medical history between patients with extreme periodic fever, aphthous stomatitis, pharyngitis, and cervical adenitis (ePFAPA) and non-extreme PFAPA (nPFAPA)

Variables	ePFAPA	nPFAPA	P value
Male sex, n (%)	31/47 (66%)	173/318 (54%)	0.14
Mediterranean ancestry, n (%)	21/42 (50%)	149/299 (50%)	0.98
Sephardic, n (%)	20/42 (48%)	105/299 (35%)	0.12
Arabic, n (%)	1/42 (2%)	44/299 (15%)	0.027
Ashkenazi ancestry, n (%)	1/42 (2%)	12/299 (4%)	> 0.99
Multiethnic ancestry, n (%)	20/42 (48%)	138/299 (46%)	0.86
Consanguinity, n (%)	0 (0%)	6/296 (2%)	> 0.99
Family history of presumed PFAPA*, n (%)	15/44 (34%)	95/298 (32%)	0.76
Family history of FMF, n (%)	14/43 (33%)	66/304 (22%)	0.11
Family history of tonsillectomy, n (%)	5/28 (18%)	25/203 (12%)	0.39

* Based on a familial history of recurrent fevers with signs of aphthous stomatitis, pharyngitis, and cervical adenitis in at least one first-degree relative

FMF, familial Mediterranean fever

The ePFAPA group comprised 47 patients. The nPFAPA group comprised 318 patients. The proportions and the percentages reflect the missing data

Data were available for 341 patients. Of them, 170 (50%) were of Mediterranean ancestry (i.e. Israeli Arabs and Sephardic Jews) and 13 patients (4%) were Ashkenazi Jews. The proportion of Arabs in the ePFAPA group was low compared to the nPFAPA group (2% vs. 15%, P = 0.027). The family histories of presumed PFAPA, FMF, and tonsillectomy were similar between the groups (Table 1).

Table 2 shows the clinical features of the ePFAPA and nPFAPA groups. For patients with ePFAPA compared to nPFAPA, the median (IQR) age was younger at first presentation: 1.5 (0.7–2.5) vs. 2.5 (1.5-4.0) years, P < 0.001, and at diagnosis: 2.6 (2.0–3.6) vs. 4.5 (3.0–6.2) years, P < 0.001.

**Table 2:** Comparison of clinical features between patients with extreme periodic fever, aphthous stomatitis, pharyngitis, and cervical adenitis (ePFAPA) and non-extreme PFAPA (nPFAPA)

Variables	ePFAPA	nPFAPA	P value
Age at symptom onset (years), Median (IQR), N = 329	1.5 (0.7–2.5)	2.5 (1.5-4.0)	< 0.001
Age at diagnosis (years), Median (IQR), N = 3 18	2.6 (2.0–3.6)	4.5 (3.0–6.2)	< 0.001
Duration of flare (days), Median (IQR), N = 239	3.5 (2.5–5.0)	4.0 (3.0–5.5)	0.86
Initial interval between flares (weeks), Median (IQR), N = 330	2.0 (1.5–3.5)	4.0 (2.5–4.5)	< 0.001
Intervals between flares after initiation of abortive treatment with corticosteroids (weeks), Median (IQR), N = 192	1.0 (1.0–1.5)	2.5 (1.9–4.9)	< 0.001
Maximal fever (°C), Median (IQR), N = 134	40.0 (39.6–40.0)	40.0 (39.2–40.0)	0.35
Pharyngitis (%)	44/47 (94%)	290/309 (94%)	> 0.99
Adenitis (%)	24/47 (51%)	112/307 (36%)	0.056
Aphthous stomatitis (%)	13/47 (28%)	72/309 (23%)	0.51
Abdominal pain (%)	23/47 (49%)	142/309 (46%)	0.7
Headache (%)	6/47 (13%)	45/308 (15%)	0.74
Myalgia (%)	15/47 (32%)	79/308 (26%)	0.36
Arthralgia (%)	8/47 (17%)	50/308 (16%)	0.89
Rash (%)	1/47 (2%)	14/318 (4%)	0.7

IQR, interquartile range

The ePFAPA group comprised 47 patients. The nPFAPA group comprised 318 patients

The proportions and the percentages reflect the missing data

The duration of flares and maximal fever documented were similar between the groups. However, among the patients with ePFAPA, the median (IQR) of the interval between flares upon presentation was shorter than among the patients with nPFAPA: 2.0 (1.5–3.5) vs. 4.0 (2.5–4.5) weeks, P < 0.001. Other than fever, pharyngitis was the most common symptom in both groups (94%). Patients with ePFAPA had a higher tendency to present with adenitis; however, these numbers were not sufficiently powered to achieve statistical significance (51% vs. 36%, P = 0.056). Other presenting symptoms were reported by the two groups at a similar rate: almost half the patients in both groups had abdominal pain during flares and about one quarter had aphthous stomatitis. Myalgia was reported by 32% in the ePFAPA group and by 26% in the nPFAPA group, whereas headache and arthralgia were reported at lower rates.

Table 3 shows the various prophylactic treatments the patients received based on the providers’ discretion. Higher proportions of patients in the ePFAPA than the nPFAPA group received prophylactic treatment with colchicine (53% vs. 19%, P < 0.001) and montelukast (15% vs. 3%, P < 0.001). The proportion of patients who underwent tonsillectomy was slightly less in the ePFAFA than the nPFAFA group, although the small absolute number makes this difference negligible. Among patients with ePFAPA, responses to prophylactic treatment were similar between those who met the criteria of this group at first presentation and those who met the criteria after corticosteroid treatment. The only exception was a better response to colchicine in the former than the latter (90% vs. 47%, P = 0.04). However, the absolute numbers are too small (9 vs. 7) to draw conclusions.

**Table 3:** Comparison of prophylactic treatment response between patients with extreme periodic fever, aphthous stomatitis, pharyngitis, and cervical adenitis (ePFAPA) and non-extreme (nPFAPA)

Variables		ePFAPA (N = 47)	nPFAPA (N = 318)	P value
Colchicine	Patients treated, n (%)	25 (53%)	61 (19%)	< 0.001
	Response (no/fewer flares), n (% of treated)	16 (64%)	43 (70%)	0.56
Cimetidine	Patients treated, n (%)	3 (6%)	6 (2%)	0.13
	Response (no/fewer flares), n (% of treated)	0 (0%)	2 (33%)	0.50
Montelukast	Patients treated, n (%)	7 (15%)	11 (3%)	< 0.001
	Response (no/fewer flares), n (% of treated)	2 (29%)	4 (36%)	> 0.099
Tonsillectomy	Patients treated, n (%)	1 (2%)	12 (4%)	> 0.99
	Response (no/fewer flares), n (% of treated)	1 (100%)	3 (25%)	0.31

Twenty-two (47%) of the patients with ePFAPA already had extreme PFAPA, with intervals of less than two weeks upon the first presentation with PFAPA. After treatment with steroids, 25 (53%) became extreme PFAPA. These two subgroups of the ePFAPA are compared in Tables 4 and 5. Among the patients who met ePFAPA criteria at first presentation compared to those who met the criteria after corticosteroid treatment, the median duration of febrile attacks was shorter: 3 days (2.5–3.75) vs. 4.5 days (3.5–6.75), P = 0.047. Other clinical and demographic variables, including the young age at presentation, were similar between the subgroups of ePFAPA.

**Table 4:** Comparison of demographic data and family medical history of patients with extreme periodic fever, aphthous stomatitis, pharyngitis, and cervical adenitis (ePFAPA), between those who met these criteria after steroid treatment (erPFAPA) and those who met the criteria upon first presentation

Variables	erPFAPA	ePFAPA upon presentation	P value
Male sex, n (%)	15/25 (60%)	16/22 (73%)	0.36
Mediterranean ancestry, n (%)	9/24 (38%)	12/18 (67%)	0.06
Sephardic, n (%)	9/24 (38%)	11/18 (61%)	0.13
Arabic, n (%)	0 (0%)	1/18 (5.6%)	0.43
Ashkenazi ancestry, n (%)	0 (0%)	1/18 (5.6%)	0.43
Multiethnic ancestry, n (%)	15/24 (62%)	5/18 (28%)	0.03
Consanguinity, n (%)	0 (%)	0 (0%)	
Family history of presumed PFAPA*, n (%)	8/24 (33%)	6/20 (30%)	0.81
Family history of FMF, n (%)	9/24 (38%)	5/19 (26%)	0.44
Family history of tonsillectomy, n (%)	2/16 (12%)	3/12 (25%)	0.62

* Based on a familial history of recurrent fevers with signs of aphthous stomatitis, pharyngitis, and cervical adenitis in at least one first-degree relative

FMF, familial Mediterranean fever

The erPFAPA group comprised 25 patients. The ePFAPA group upon presentation comprise 22 patients. The proportions and the percentages reflect the missing data

**Table 5:** Comparison of clinical features of patients with extreme periodic fever, aphthous stomatitis, pharyngitis, and cervical adenitis (ePFAPA), between those who met these criteria after steroid treatment (erPFAPA) and those who met the criteria upon first presentation

Variables	erPFAPA	ePFAPA upon presentation	P value
Age at symptom onset (years), Median (IQR), N = 41	1.67 (1.0–2.5)	1.5 (0.5–2.25)	0.42
Age at diagnosis (years), Median (IQR), N = 44	2.25 (2.0–3.38)	3.0 (1.5–4.25)	0.75
Duration of flare (days), Median (IQR), N = 29	4.5 (3.5–6.75)	3.0 (2.5–3.75)	0.047
Initial interval between flares (weeks), Median (IQR), N = 41	3.5 (3.0–4.0)	1.5 (1.0–2.0)	< 0.001
Intervals between flares after initiation of abortive treatment with corticosteroids (w), Median (IQR), N = 36	1 (1.0–1.5)	1 (1–1.38)	0.32
Pharyngitis (%)	23/25 (92%)	21/22 (95%)	> 0.99
Adenitis (%)	12/25 (48%)	12/22 (55%)	0.65
Aphthous stomatitis (%)	7/25 (28%)	6/22 (27%)	0.96
Abdominal pain (%)	13/25 (52%)	10/22 (45%)	0.65
Headache (%)	4/25 (16%)	2/22 (9.1%)	0.67
Myalgia (%)	8/25 (32%)	7/22 (32%)	> 0.99
Arthralgia (%)	5/25 (20%)	3/22 (14%)	0.71
Rash (%)	1/25 (4%)	0 (0%)	> 0.99
Colchicine response	7/15 (47%)	9/10 (90%)	0.04

The erPFAPA group comprised 25 patients. The ePFAPA group upon presentation comprise 22 patients. The proportions and the percentages reflect the missing data

All the patients were treated with abortive corticosteroids and their febrile attacks subsequently resolved. Of the entire study population for whom data were available, for 103 (46%) patients, the interval between flares shortened after steroid treatment. In subgroup analysis (Table 6), the interval shortened more among those who met ePFAPA criteria after steroid treatment than at first presentation (P = 0.002). The interval between flares shortened more for the ePFAPA than the nPFAPA group: 1 (1–1.5) vs. 2.5 (1.9–4.0) weeks, P < 0.001.

**Table 6:** Comparison of the interval between flares after steroid treatment between patients with non-extreme periodic fever, aphthous stomatitis, pharyngitis, and cervical adenitis (nPFAPA), extreme responder PFAPA(erPFAPA) and extreme PFAPA upon presentation (ePFAPA upon presentation)

Variables	nPFAPA (N = 318)	erPFAPA (N = 25)	ePFAPA upon presentation (N = 22)	P value
Increased episode frequency with steroids, n (%)	74/183 (40%)	20/24 (83%)	9/16 (56%)	0.002
Intervals between flares after initiation of abortive treatment with corticosteroids (weeks), Median (IQR), N = 192	2.5 (1.93–4)	1.0 (1.0–1.5)	1.0 (1.0–1.38)	< 0.001

The nPFAPA group comprised 318 patients. The erPFAPA groupcomprised 25 patients. The ePFAPA group upon presentation comprise 22 patients. The proportions and the percentages reflect the missing data

**Fig. 1:**
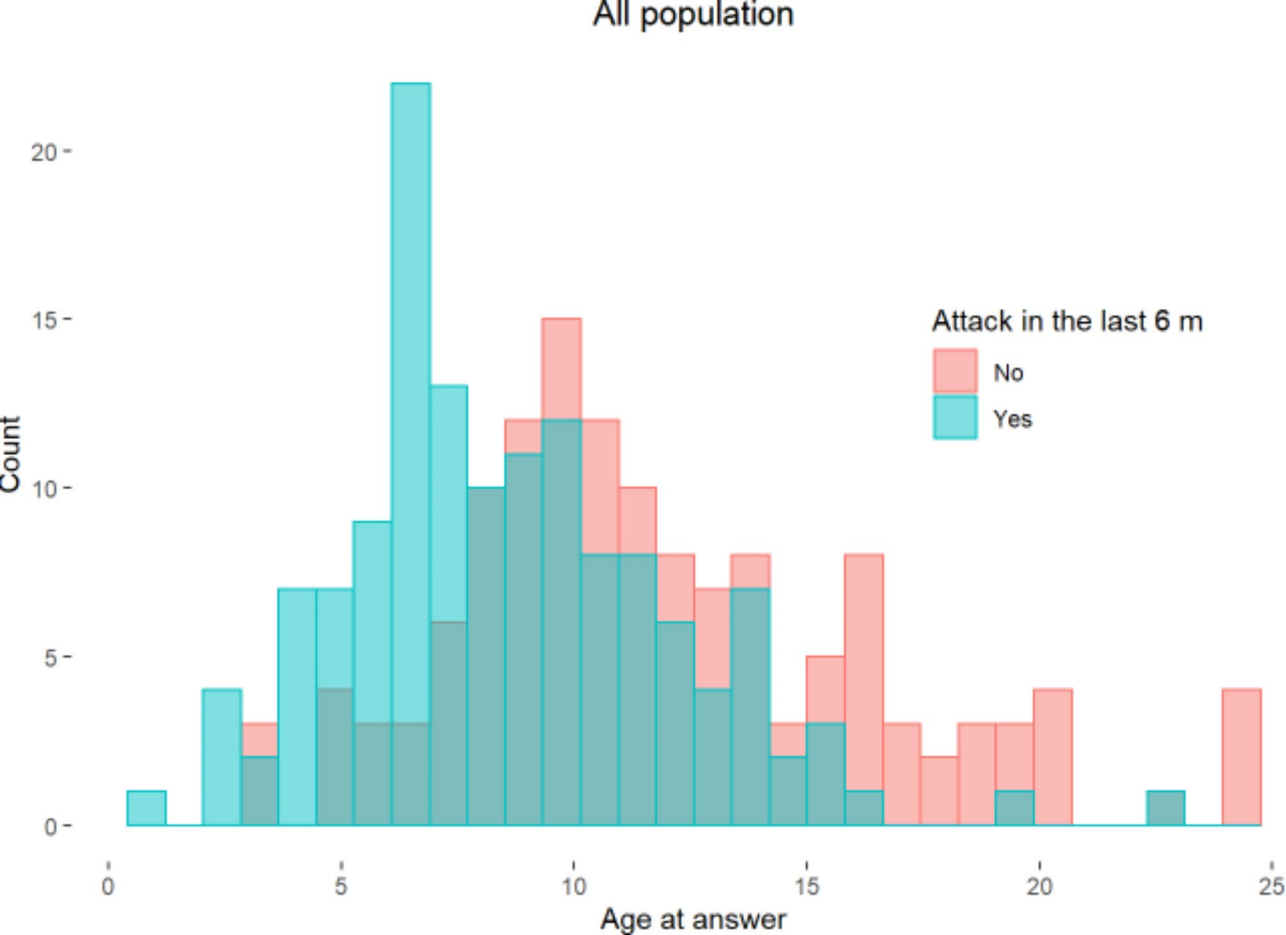
Results of a telephone survey in which the patients or their parents were asked whether they had an attack in the preceding 6 months

Figure 1 shows the results of the survey regarding the activity of the disease. Parents of 238 (75%) patients from the nPFAPA group (mean age 10.6 years) and of 39 (83%) patients from the ePFAPA group (mean age 8.3 years) answered the survey. Regardless of group classification, about 95% of all the patients whose parents responded, did not have attacks after age 14 years.

## 2. Discussion

This study describes for the first time a subgroup of patients with PFAPA who presented with short intervals between flares (less than two weeks apart) and who were hence classified as having extreme PFAPA. The analysis allowed a period of at least 3 consecutive months of frequent flares (at any time period since disease onset) to avoid potential biases such as recall bias. This group comprised 12.9% of all our patients with PFAPA. However, their proportion in the overall Israeli PFAPA population may be even lower, due to possible underdiagnosis of PFAPA, mainly among individuals with relatively mild disease and infrequent flares. Of note, unlike previous reports, none of our patients showed clockwise intervals.

Among the patients with ePFAPA, febrile attacks were shorter among those who met these criteria at first presentation than among those who met the criteria after corticosteroid treatment (3 days (2.5–3.75) vs. 4.5 days (3.5–6.75), P = 0.047). None of the other characteristics examined differed between these subgroups of ePFAPA.

Considering the impact of flares of PFAPA on quality of life and the evidence that quality of life decreases as flares become more frequent [13], we hereby discuss both groups as one entity, namely ePFAPA. About half our patients with PFAPA, both ePFAPA and nPFAPA, were of Mediterranean descent (i.e. both parents were of Mediterranean ancestry), consistent with our previous study [9]. Indeed, Batu et al. found that compared to American patients with PFAPA, among patients of Turkish descent, the duration of fever attacks was shorter, in addition to an earlier age of onset [11]. Although the proportion of Israeli Arabs was lower in the ePFAPA than the nPFAPA group (2% vs. 15%, P = 0.027), the numbers are too small to draw conclusions. Family history of presumed PFAPA was about 30% in both groups, which is higher than previously reported [6, 18].

An important finding of this study is the observation that among patients with ePFAPA, the disease started at a younger age and diagnosis was earlier. Also, the median interval between flares at disease onset was shorter for these patients than for patients with nPFAPA: 2.0 (1.5–3.5) vs. 4.0 (2.5–4.5), P < 0.001. Lastly, the routine treatment of PFAPA with a single dose of corticosteroids resulted in a significant increase in the frequency of flares for 46% of the study population (for whom data were available). This rendered the median interval between flares of ePFAPA even shorter than that of patients with nPFAPA: 1 (1–1.5) vs. 2.5 (1.9–4.0) weeks, P < 0.001. This is consistent with studies that showed that steroids shorten the interval between flares in about half the patients with PFAPA [12].

Considering all the above, a patient with PFAPA who presents with a relatively high frequency of flares at a younger age than usual should be considered as potentially having ePFAPA. This should prompt closer follow up, for they are prone to have a higher frequency of flares, particularly after the introduction of treatment with abortive corticosteroids. Moreover, for these patients, treatment with prophylactic agents such as colchicine might be considered earlier in the course of the illness. Indeed, 53% of our patients with ePFAPA were eventually treated with colchicine (cimetidine is not commercially available in Israel) compared to only 19% of the patients with nPFAPA (P < 0.001), although the response rate to colchicine among the patients who were treated was similar between the groups (64% and 70%, respectively, P = 0.56). This response rate is comparable to rates reported by others [2, 4, 9, 10]. Notably, tonsillectomy was shown to be the most effective intervention for long-term resolution of PFAPA [19]. The low rate of tonsillectomy among our patients is consistent with the considerably lower frequency of tonsillectomy for PFAPA resolution in Israel than in the USA [20]. Lastly, many patients with PFAPA eventually experience spontaneous symptom resolution without sequelae [4]. Indeed, our survey showed that the vast majority of our patients with PFAPA did not have attacks after age 14 years, regardless of their group classification.

As PFAPA improves with age, it may also present with shorter intervals at early ages, as a result of changes in the immune system with age [11]. This might explain the presentation of ePFAPA at an earlier age than nPFAPA. The observation of no other difference between these two groups also supports this notion. Of importance, FMF and PFAPA are two distinctive entities, and one of them can evolve into the other [10]. As a result, for ethical reasons, patients in our center with PFAPA do not undergo routine genetic testing for FMF (mutations in the MEFV gene). Thus, genetic data are available only from external resources or if at any point FMF disease is suspected. Therefore, due to a possible selection bias, comparing MEFV mutations in this cohort is irrelevant.

Apart from its retrospective nature, the main limitation of our study is the relatively small number of patients with ePFAPA. In addition, a referral bias might be relevant as patients referred to our clinic may experience a higher-than-average frequency of flares. This would have resulted in a higher proportion of patients with ePFAPA than in the general population. The retrospective nature of the study did not enable analysis of the duration in which the ePFAPA group remained with high frequency of fever attacks. Moreover, a much higher proportion of patients with ePFAPA received colchicine. This may have resulted in a bias regarding the treatment response. Furthermore, we only have information regarding flare frequency after steroids for two-thirds of our patients. This raises the possibility of a selection bias, presumably due to resolution of the disease among patients who were lost to follow-up. Finally, although patients were usually asked to continually fill a “flare diary”, recall bias cannot be ruled out. To mitigate recall bias, in the survey we conducted in April 2021, we inquired only whether the disease was still active in the preceding 6 months.

## Conclusions

This study is the first documentation of PFAPA with short intervals between flares of less than two weeks. This study shows, that in patients with frequent flares at presentation, flare frequency might be increased even further after introduction of abortive treatment with corticosteroids. Therefore, these patients may require closer follow-up, including earlier initiation of prophylactic treatment for PFAPA flares or tonsillectomy, for they are more likely to become extreme PFAPA.

### Electronic supplementary material

Below is the link to the electronic supplementary material.

Supplementary Material 1

## Data Availability

See Tables 1, 2, 3, 4, 5 and 6, pages 15–22.

## Abbreviations

PFAPA: Periodic fever, aphthous stomatitis, pharyngitis, adenitis
ePFAPA: Extreme PFAPA
nPFAPA: Non-extreme PFAPA
FMF: Familial Mediterranean fever
