# Supplementary material for: Extreme Periodic Fever, Aphthous Stomatitis, Pharyngitis, Adenitis (PFAPA): a discrete group of patients
Source: Pediatr Rheumatol Online J. 2023 Sep 1;21:93. doi: 10.1186/s12969-023-00880-1 (PMC10474755; doi:10.1186/s12969-023-00880-1)
Supplement: Supplementary file 1 — Supplementary Material 1 [file 12969_2023_880_MOESM1_ESM.docx]

PROJ-D-23-00098
Extreme Periodic Fever, Aphthous Stomatitis, Pharyngitis, Adenitis (PFAPA): A Discrete Group of Patients

**General comment by the authors** - We would like to thank the four reviewers who dedicated their time and expertise to review our manuscript. We carefully considered and responded to every comment and question, and believe our revised manuscript is more accurate, coherent and interesting subsequent to the feedback. Thank you for the opportunity to improve and enrich our study.

**Reviewer #1:**

The authors created a subset of patients (e-PFAPA) in the PFAPA group who had a flare of fewer than two weeks between attacks and compared it with other n-PFAPA groups. I think that this categorization seems to be both novel and that the term (e-PFAPA) is not widely accepted.

**Answer: Thank you for your comment.**

It is well known that the disease resolves completely after the age of 14. In other words, while it improves with age, it can be expected to show a different clinical course with shorter intervals at early ages. This may be the result of changes in the immune system with age. Therefore, instead of saying extreme PFAPA, it can be claimed that the attack interval may be shorter for those who start at an early age. I think it would be appropriate to discuss this issue in the Discussion section. There was no difference between these two groups, which may also lend support to this notion. Therefore, it would be more accurate to state that the early age of onset of PFAPA may result in short intervals between attacks.

**Answer: This is an important insight, which we have added to the Discussion section: "As PFAPA improves with age, it may also present with shorter intervals at early ages, as a result of changes in the immune system with age [11]. This might explain the presentation of ePFAPA at an earlier age than nPFAPA. The observation of no other difference between these two groups also supports this notion."**

In this cohort, however, it would be appropriate to construct subgroups according to the onset age of the PFAPA and compare them in terms of attack intervals.

**Answer: Thank you for this suggestion. We tried to create a sub analysis based on age onset. However, this was not possible due to an age difference of less than one year between the two groups (median of 1.5 vs 2.5 in the ePFAPA and nPFAPA groups respectively).**

**Reviewer #2:**

Thank you for submitting your report, it was very interesting, especially the results that apart from the frequency there did not seem to be a difference in percentages of presenting symptoms among ePFAPA/nPFAPA. That could argue for an unrelated etiology of the fevers or the ePFAPA group could have a different IL-1 signature/pathway defect. 
Obviously, there is a lot of interest in PFAPA especially when presentation is less typical. This is a large cohort and there might be more information in the data.

**Answer: Thank you very much for the positive feedback.**

Also, I found the labeling of ePFAPA for both at onset/post steroid therapy increased frequency confusing, maybe one could consider an er(extreme responder)PFAP or xPFAPA for those responding to steroids with a shortening of the interval and keep the ePFAPA for the high-frequency-flare-at-onset group.

**Answer: Thank you for this feedback. We changed the labeling accordingly: "Hence, the definition of ePFAPA included both patients who started their disease with short intervals (ePFAPA upon presentation) and patients who met the criteria for ePFAPA during the course of their disease. The latter occurred after initiation of a single dose of treatment with glucocorticoids during an attack (erPFAPA, extreme responder PFAPA). In a sub-analysis, we compared characteristics between the three groups: nPFAPA, ePFAPA upon presentation, and erPFAPA".**

A few comments regarding:
A. Content:1. As for the studied group: PFAPA definition - you mention using the newer Vanoni 2018 listed criteria. How were infections, immunodeficiency, and cyclic neutropenia excluded? Specifically, were patients with URI symptoms excluded? How did you exclude patients with FMF? Did all of them have genetic testing for FMF, MVK, and TRAPS? Based on prior publications from the area, there seems to be an increased prevalence of FMF mutations even among PFAPA patients published by Dr. Padeh. The clockwork periodicity is actually a cardinal feature of PFAPA, so I can not agree with the comment in the introduction:  "Occasionally, flares may occur at predictable intervals (Federici et al., 2014)." What percentage of patients did have such periodicity and was there a difference between them re: presenting symptoms/length of symptom?

**Answer: Thank you for these important comments.**

**Patients with infections, immunodeficiency and cyclic neutropenia were excluded based on their medical histories, physical examinations and lab work. Patients with URI symptoms were excluded based on classical PFAPA symptoms (pharyngitis, aphtha, etc.) and, more specifically, the resolution of fever within hours after treatment with a single dose of steroids. We added a paragraph on this topic to the Methods section: "A fourth criterion is exclusion of patients with other causes of recurrent fever, infections, immunodeficiency and cyclic neutropenia, according to their medical histories, physical examinations and lab work". Later, we wrote: "PFAPA attacks were identified according to classical symptoms and complete resolution of fever within hours of steroid treatment. Responses to steroids and symptoms differentiated between PFAPA flairs and upper respiratory tract infections."**

**PFAPA and FMF are both prevalent in Israel. Their clinical presentations differ** substantiall**y. Hence, patients who presented with classical PFAPA attacks, as evident from their symptoms, the duration of fever and their clinical response to steroids (the latter together with a lack of APA symptoms distinguished PFAPA from FMF), did not undergo genetic testing for FMF. An exception was those who later developed classical FMF attacks; these were excluded from the analysis.**

**MVK and TRAPS are rare diseases with specific manifestations. As they differ from PFAPA, individuals with PFAPA do not routinely undergo genetic evaluations for such diseases.**

**While clockwork periodicity has high specificity, it has low sensitivity. Indeed, none of our patients showed fixed intervals of attacks. We added this information to the discussion: "Of note, unlike previous reports, none of our patients showed clockwise intervals".**

2. Why was the median for the interquartile range chosen instead of the median for the whole group?

**Answer: Thank you for noticing this. We did not clarify this point. The median we used was for the entire group. We made the appropriate corrections in the revised manuscript.**

Choosing the interval under 2 weeks for ePFAPA is valid, nevertheless, most patients will be in the 3-5 week range and the 2 weekers could be already outliers, was there a sharp difference between ePFAPA group and the "2 - weekers"? Do your numbers differ if you include the 2 weekers? I am just trying to see if the less than 3-week group is actually an ePFAPA population, rather than a less than 2-week group.

**Answer: Thank you for this important comment. We tried to perform an analysis for intervals shorter than 3 weeks, but there were no statistically significant differences. For this reason, and to emphasis the liability of frequent fevers on families and health care providers, we decided to use a cutoff of 2 weeks.**

3. Higher colchicine prophylaxis for ePFAPA patients is listed - was it based on the provider's discretion?

**Thank you for this comment. It was indeed based on the providers' discretion. We have added this to the Results section: "Table 3 shows the various prophylactic treatments the patients received based on the providers' discretion"**

4. The abstract's conclusion is confusing:  "About half the patients categorized with ePFAPA syndrome met the criteria upon presentation with PFAPA". How was the other half of the ePFAPA group diagnosed with PFAPA then?

**Answer: Thank you for this important comment; the meaning was that half the patients with ePFAPA already had extreme PFAPA features upon presentation (in contrast to those with erPFPAA, who presented with extreme features after treatment with steroids). We clarified this point in the Abstract: "About half the patients categorized with ePFAPA syndrome already had extreme features upon presentation".**

5. In the introduction, you mention the genetic predisposition for PFAPA, which is likely true and PFAPA likely has a polygenic etiology. The group of  Mediterranean ancestry needs some clarification, it seems, in the results the Israeli Arabs are separate from the Mediterranean group but in the tables, they do belong to that. Could you please, clarify this? Your argument for the Mediterranean "factor" in genetics is based on Amarilyo 2020 report, which has a  selection bias there due to the origin of the analyzed population. In that report, it seems Sephardic Jewish and Arabic descent were counted as Mediterranean, and obviously, there are other ethnicities in the Mediterranean region. Could you clarify who you count as having Mediterranean ancestry? It looks like mostly Sephardic Jews, right?

**Answer: Thank you for this question. In our study, Mediterranean ancestry consisted only of Sephardi Jews and Israeli Arabs (there are almost no other Mediterranean ancestries in Israel, and specifically, not in our cohort). The error in the results was corrected: "Data were available for 341 patients. Of them, 170 (50%) were of Mediterranean ancestry (i.e. Israeli Arabs and Sephardic Jews) and 13 patients (4%) were Ashkenazi Jews".**

6. In results - what triggered tonsillectomy? As for colchicine response being better in ePFAPA, it seems, a much higher proportion of patients in ePFAPA received colchicine, to begin with, was that accounted for in the response? If it is so, it could be clinically relevant to reach for colchicine in ePFAPA patients faster than in nPFAPA.

**Answer: Thank you for these questions.**

**We do not routinely recommend tonsillectomy. Following this comment, we reviewed the medical records of the few patients who underwent tonsillectomy. For two of 13 of them, recurrent tonsillitis was the reason specified for the procedure. Indications were not recorded for the remaining patients.**

**The higher proportion of colchicine treatment in the ePFAPA group might result in a bias. We added this point to the Discussion section under the limitation paragraph: "Moreover, a much higher proportion of patients with ePFAPA received colchicine. This may have resulted in a bias regarding the treatment response".**

**We mitigated this bias by analyzing the response to treatment only among the patients who were treated with colchicine in both groups (% of treated – see the highlight in table 3). No significant differences were seen. In the discussion section, we suggested that earlier introduction of colchicine might be warranted.**

7. Again, in the results, could you please clarify:  "Twenty-two (47%) of the patients with ePFAPA filled the criteria of ePFAPA upon first presentation with ePFAPA".

**Answer: Thank you again, we corrected the paragraph as follows: "Twenty-two (47%) of the patients with ePFAPA already had extreme PFAPA, with intervals of less than two weeks upon the first presentation with PFAPA. After treatment with steroids, 25 (53%) developed extreme PFAPA".**

8. In the discussion part,  I do not think you have the data/numbers to recommend cimetidine for ePFAPA.

**Answer: Thank you for this comment, with which we agree (Cimetidine is not commercially available in Israel). We removed the statement from the discussion section.**

9. In the discussion part, the paragraph on FMF and PFAPA link is confusing- prior reports coming from your area have listed the increased presence of FMF mutations/variants in PFAPA patients. Is that the same percentage as in the general population with no FMF? What do you mean under " ethical reasons" not to test for FMF? Table 1 does seem to bring up the point of FMF in families. You reported earlier that FMF patients were indeed excluded, but here you argue that it is irrelevant to look for MEFV mutations. Once again, how did you exclude FMF patients then? It also sounds like you argue, that FMF patients can evolve into PFAPA? I can see it occurring the other way due to the scarcity of symptoms at onset; how many of your 365 PFAPA patients "have evolved" into FMF? Having MEFV testing for all PFAPA patients in your area specifically could point to a subgroup of PFAPA patients who would require closer monitoring and checking inter-flare SAA levels. 

**Answer: Thank you for these important comments. As we mentioned in response to section A1, there are significant clinical differences between PFAPA and FMF. A main difference is the presentation of pharyngitis and the complete resolution of fever in PFAPA, in response to steroids. Patients whose symptoms evolved to classical FMF attacks including unresponsiveness to steroids underwent genetic testing and were excluded from the study regardless of their genetic mutations (because they were clinically diagnosed with FMF). We do not have data of the proportions of patients who were excluded for this reason. However, in the Journal of Pediatrics (2019) using a similar cohort, Butbul et al. reported that 18.9% of patients with PFAPA developed FMF Therefore, we assume that our numbers are similar.**

**To clarify the ethical reason, if we would test all the individuals with PFAPA and identify two FMF mutations, this would not reveal if and when FMF would present. The information would not change the management of patients who present clinically with PFAPA attacks. Such patients are usually given a single dose of steroids to halt attacks unless the attacks become frequent. The genetic detection of FMF in the absence of clinical symptoms would only cause stress to patients and their families. Hence, our clinic's approach is to test for FMF only for patients who present with FMF-like attacks (including unresponsiveness to steroids).**

B. Tables and Figures:

3. Table 6 – The P value is comparing which 2 of the 3 groups?

**Thank you for this question. The P value is comparing the three groups.**

**Reviewer #3:**

It is good to see to describing first time a subgroups of patients diagnosed with PFAPA. Auto-inflammatory clinical conditions should be defined in more detail in order to shed light on the diagnosis and treatment for clinicians. This article should be accepted.

**Answer: Thank you for the positive feedback.**

**Reviewer #4:**

This manuscript presents a retrospective cohort of patients with PFAPA from a single institution and characterizes similarities and differences between those with fever interval of <2 weeks (ePFAPA) and those with fever interval >2 weeks (nPFAPA). The study limitations identified by the authors include a small sample size for the ePFAPA group and potential for recall bias.  Within these limitations, the authors find that characteristics between the two groups are overall similar, apart from age of onset and diagnosis, use of prophylactic medications and response of fever interval to abortive corticosteroid initiation. 

The study is of interest as differentiating subgroups of patients with PFAPA may inform about management options, particularly with respect to what may be expected with corticosteroids or prophylactic medication trials.

The manuscript is well-written, clear and concise.

**Answer: Thank you very much for the kind words.**

I have the following questions/comments for the authors:

1) Please comment on how the 365 patient records were chosen for this study.  Does this represent all patients with PFAPA at the institution during this period?

**Answer: These are indeed all the patients with PFAPA who were treated in our clinic during the study period. We added this to the methods section: "Data were collected retrospectively from the electronic medical charts of all the children diagnosed with PFAPA who were followed at the outpatient pediatric rheumatology clinic of Schneider…"**

2) How was missing data handled for the analysis?

**Answer: Thank you for this question. Missing variables were omitted from the final calculations after verifying that their omission would not cause clinical or statistical bias. We added this point to the methods section: "Missing variables were omitted from the final calculations after verifying that this would not cause clinical or statistical bias".**

3) The requirement to meet the diagnosis of ePFAPA was a fever interval of <2 weeks for at least 3 months during any period of the study. Does the dataset allow for identification of how dynamic this was (ie. did individuals with ePFAPA tend to stay as ePFAPA, or are there patients who had transient periods of increased frequency that may be classified within the ePFAPA group)?

**Answer: Thank you for this important question. Unfortunately, we do not have this information due to the retrospective nature of the study. We have added this to the limitations of the study: "The retrospective nature of the study did not enable analysis of the duration in which the ePFAPA group remained with high frequency of fever attacks".**

4) With respect to the classification criteria, please comment on why this set was used rather than the subsequently developed EULAR/PRINTO criteria.  Also, does 'complete recovery' include normalization of inflammatory markers?

**Answer: Thank you for this important question. After thorough review, our patients met both EULAR/PRINTO criteria and Vanoni criteria. We added this point to the methods section: "The diagnosis of PFAPA was based on previously established clinical criteria (Vanoni et al 2018, Gattorno et al 2019)".**

**As to complete recovery, we do not check inflammatory markers routinely once febrile attacks resolve. This is because PFAPA is in nature a transient pediatric illness and inflammatory marker levels are high and nonspecific.**

5) With respect to treatment response, what duration of treatment was required to count as an adequate trial?  How was failure due to intolerance handled in the analysis?

**Answer: Thank you for this question. An adequate trial was based on the providers' discretion. We generally used Betamethasone, which is palatable, so intolerance is infrequent. The minority of patients who complained of behavioral changes were switched to Prednisolone. The only "side effect" was interval shortening, which we addressed.**

6) Was there a difference in steroid responsiveness between the two groups?

**Answer: All the children were treated with steroids, with consequent resolution of fever. This helped differentiate between PFAPA attacks and fever due to infection. We added this point to the Results section: "All the patients were treated with abortive corticosteroids, and their febrile attacks subsequently resolved."**
7) It appears that only about 2/3 of patients had data regarding fever frequency following corticosteroids.  Could the authors comment on whether the observed differences in interval shortening may reflect biases in data collection?

**Answer: Thank** **you for this important insight. One-third of the patients were indeed lost to follow up. We speculate that they probably "outgrew" PFAPA and hence stopped their clinical visits. Thus, the remaining two-thirds reflect the most "active" patients with PFAPA. This raises the possibility of a bias. We added this to the limitations of the study: "Furthermore, we only have information regarding flare frequency after steroids for two-thirds of our patients. This raises the possibility of a selection bias, presumably due to resolution of the disease among patients who were lost to follow-up".**
